# Supposed Dialogue about Temperance

**Published:** 1885-11

**Authors:** 


### Supposed Dialogue about Temperance.

I propose in this paper to present what I consider a great, if not the greatest, hinderance against the efforts of the Church and her missionaries in convincing the world of the divine truth of our holy religion.

The Christian missionary calls upon one or more of these sages and invites them to abandon their religion and embrace ours. The sage replies : "I do not see that your religion curbs the passions of her votaries or makes them even decent men. Your papers chronicle crime, assaults, and most horrid murders committed daily by your people."

C. M. replies : "These crimes are committed by persons crazed by the use of intoxicating drinks, and are hardly responsible for their acts."

S. "But your laws lisence and make the sale of these drinks legal, appoint officers to locate them, and armed police to protect them in their wickedness."

C. M. "These laws are not placed upon our statue books by Christians."

S. "But surely neither the Mohammedans, Parsees, Buddhists, or even the Jews, have done this. Yours is a Christian country, and Christians alone are guilty."

C. M. "What I mean is that the professors of religion, the ministers and members of our churches, have not done it, but men who neither fear God nor man. You have the same class among you "

S. "True, we have wicked men that commit crimes, but our magistrates punish them for such offenses ; but your laws actually establish these criminal manufactories, and become a party in their criminal acts."

C. M. "You surely will not hold our holy religion responsible for every bad law you find on the statute in Christian countries ? "

S. "Why, your Bible says wisely, 'By their fruits ye shall know them ;' a people never rises higher or are better than the laws they enact. I am aware that yours is a republic, and the majority must rule ; it is very clear to my mind that your majorities must be very bad men to vote for the election of legislators who are capable of enacting such injudicious laws. But do your good men combine and unite together, and by their votes endeavor to elect better men ? "

C. M. "They have of late to a limited extent."

S. "To a limited extent? Am I, then, to understand that most of your ministers, church-members, and what you call good men, aid by their votes to elect these legislators? I must, then, logically hold your people, as an entirety, as sustaining, in their most effectual way, unrighteous traffic. Whatever reason your people may have to allow those laws to remain on your statue books, creating poverty and criminals among yourselves, our people, our legislators, our laws, and our religion, all protest against you authorizing these beverages to be carried in your ships, brought into our country, to seek to decoy, to tempt and to turn from the path of virtue and religion our own people. You are a powerful nation, and we fear you and your men-of-war, and we are obliged to submit to your injustices, but we mourn over your success in the destruction of many of our youth, and there is a cry going up to Allah to save our people from the direful scourge your laws, under your religion, are forcing upon a people that never harmed you. And if I rightly understood your religion, the Christian God has not an attribute that can take your part in the wickedness. I ask, how can I, as an honest man, call upon my people to turn from their religion to embrace your civilization, that produces such bitter fruits? I certainly find excellence in the life and precepts of Jesus Christ, and I am a tually at a loss to account for the very general and almost universal practice among Christians, sustaining by their votes these wicked and inhuman laws. My friend, return to your own countrymen and plead with them to put a stop to their sailors bringing this worse than the cholera among us. When this is done, you will have better success among our people."—*Christian Advocate.*

